# R&D Investments, Debt Capital, and Ownership Concentration: A Three-Way Interaction and Lag Effects on Firm Performance in China's Pharmaceutical Industry

**DOI:** 10.3389/fpubh.2021.708832

**Published:** 2021-10-01

**Authors:** Chih-Yi Su, Yao-Ning Guo, Kuang-Cheng Chai, Wei-Wei Kong

**Affiliations:** School of Business, Guilin University of Electronic Technology, Guilin, China

**Keywords:** R&D investment, debt capital, ownership concentration, three-way interaction effect, lag effects, China's pharmaceutical industry

## Abstract

The existing literature has yet to provide consistent evidence on the relationship between R&D investments and firm performance. The current study attempted to fill this gap in the literature by examining the effect of lag structure and the moderating role of financial governance, in terms of debt capital and ownership concentration, on the returns of R&D. Analyzing a sample of China's pharmaceutical firms from 2009 to 2018, we found that the effect of R&D upon growth begins in the second year after R&D spending and increases thereafter. There exists a vigorous debate about the choice between debt and ownership structure. To fill this gap, we proposed a three-way interactive effect. The results suggest that firms that invest heavily in R&D may achieve their highest performance when the use of debt capital and the extent of ownership concentration are both low. This study contributes to the R&D investments and financial governance literature by reconciling previous mixed evidence about the returns of R&D and the debt–equity choices on R&D investment decisions.

## Introduction

The coronavirus disease 2019 (COVID-19) pandemic underscored the importance of research and development (R&D) in the pharmaceutical industry. R&D plays a key role in responding to the COVID-19 outbreak and acts as a critical lever to ensure a sustainable and inclusive recovery while boosting the resilience of the socioeconomic system (1, 2). The pharmaceutical industry devoted $186 billion dollars to R&D expenditure in 2019 (3). The share of revenues that pharmaceutical firms invest in R&D has also grown, i.e., approximately one-quarter of their revenues (net of expenses and buyer rebates) in 2019, which is almost twice as large a share of revenue as spent in 2000 (4, 5). This share is larger than that for other innovation-based industries, such as semiconductors, technology hardware, and software (5). However, committing investments to R&D projects is risky due to the long-time horizon, the nontrivial likelihood of project failure, and associated exchange hazards (6, 7). The collective shock of COVID-19 and the challenge of rapidly responding to the pandemic offer an opportunity to reconsider the pharmaceutical R&D strategy. Therefore, it is crucial to explore how pharmaceutical firms mitigate the hazards of R&D investments on current firm performance before making an investment decision.

Although extant research has studied the relationship between R&D investments and firm performance, the existing literature has yet to provide consistent evidence on this relationship. The mixed findings about the performance impact of R&D investments can be attributed to the lack of consideration of time lag effects and the existence of contingencies that moderate the main effect (8). At present, most scholars have discussed factors such as firm size (9), industry context (10–12), country context (13, 14), and disclosure of R&D investment content (15); however, less research has been conducted from the perspective of ownership and debt structures. Investments in R&D can help to build capabilities that enhance competitive advantage (16, 17), but they are subject to serious exchange hazards that require strong governance safeguards (18, 19). According to transaction cost theory and agency theory, debt and equity are alternative governance structures for safeguarding the capital invested in a firm (19, 20), and for reducing managerial discretion and agency problems (21). However, there is no clear consensus on whether debt capital and ownership concentration better mitigate or exacerbate the hazards of R&D investments (22).

We focused on the Chinese market, which is characterized by a number of peculiarities compared to other countries. Since the beginning of the COVID-19 pandemic, China's pharmaceutical industry has made significant progress in R&D for the fight against the virus. As of 15 January 2021, out of 22 total vaccine candidates in phase 3 (or combined phase 2/3) clinical trials, six originated in China (23). The Chinese government has been deeply involved with nurturing R&D capabilities for pharmaceutical firms by creating an innovation-oriented environment. However, compared to developed countries such as the USA and Korea, China's pharmaceutical industry is still facing considerable challenges. First, China's pharmaceuticals industry is heavily fragmented. Fragmentation among pharmaceutical producers not only exacerbates problems in drug safety and quality concerns, but also makes assembling the capacity for effective R&D difficult (24, 25). More than 70% of pharmaceutical manufacturers are small–medium-sized firms with operating revenues of less than $3 million USD in China (26); hence, it is difficult for them to sufficiently support R&D with all of the necessary financial resources to pursue high-quality drug discovery. Second, principal–principal conflict, which is prevalent in emerging economies such as China, might increase the risk of R&D investments (27–29). As one of the largest pharmaceutical markets in the world, China has received increasing attention from around the world. In this context, it is of great significance to understand the pharmaceutical R&D activities and investments in China. In the context of China's pharmaceutical industry, this study aimed to provide plausible answers to the following questions: (1) What is the lagged effect of R&D investments on firm performance? (2) How do ownership and debt structures affect the return of R&D investments?

This paper is intended to contribute to the existing literature in several important ways. First, this study advances R&D management research by investigating the lagged effect of R&D investments on firm performance. In debates regarding the relationship between R&D and firm performance, the literature less considers the role of the lag structure of the returns to R&D (8). The time taken, or “time lags,” between pharmaceutical research and its translation into health improvements is receiving growing attention, especially after the outbreak of COVID-19. We found the positive effect of a 2-year lag in R&D investments on firm performance. Second, this study integrated the R&D management and financial governance literature by investigating the role of debt capital and ownership concentration on the R&D–performance relationship. Prior studies have focused on factors such as firm size, advertising activity, industry context, and country context (8), while less attention has been paid to the perspective of financial governance.

Third, this study enriches the financial governance literature by providing a finer-grained insight into how debt capital, ownership concentration, and R&D investments jointly interact to predict different outcomes. Such configurations provide insight beyond that which can be identified by direct effect relationships alone (30). The inconsistent findings on the relationship between debt capital and ownership concentration assume that they play either complementary or substitute roles (31). We found a significant three-way interaction such that firms that invest heavily in R&D achieve their highest performance when the use of debt capital and the extent of ownership concentration are both low.

Finally, this study extends research on how R&D investments and financial governance influence firm performance in developing countries. Compared to developed countries, developing countries have weak financial market infrastructure and legal systems in general (32, 33). The R&D management employed in developed countries may not be appropriate for firms in developing countries (34). However, studies on this topic have mainly focused on the USA, where firms are owned by widely dispersed shareholders (35) and the security markets are well developed.

The remainder of this paper is structured as follows. Section Literature Review and Hypotheses reviews the basic concepts underlying our theoretical arguments and presents the hypotheses developed for empirical testing. Section Data and Methodology describes the data that we used in our empirical analysis. Section Results presents the results of our analysis. Section Discussion and Conclusions concludes by presenting the implications of the study and directions for future research.

## Literature Review and Hypotheses

### R&D Investments and Firm Performance

Researchers have confirmed that R&D plays an important role in firm performance. The relationship between R&D investments and firm performance has been extensively investigated across disciplines, but the results are mixed and inconclusive (36, 37). Some studies have suggested that R&D investments can increase firm performance because of the improvement in exploitative and exploratory learning capacities, technological advancement, productivity, and market competitiveness (36, 38–43). For example, Guo, Sarkar (36) found that corporate R&D investments help firms to integrate existing knowledge and streamline their production process, thereby improving their performance. From the data of European high-tech firms, Kumbhakar, Ortega-Argilés (44) found that R&D investments can improve productivity and production quality.

Some scholars have proposed a negative relationship between R&D investments and firm performance because of the associated sky-high costs, the high uncertainty of returns, and the higher probability of failure (37, 45–49). For example, Alam, Uddin (37) studied 423 firms from 12 emerging countries and found that R&D investments are negatively associated with concurrent firm performance due to their uncertain, risky, and costly nature. Vithessonthi and Racela (48) suggested that R&D intensity is negatively correlated with firm operating performance. Pandit, Wasley (50) claimed that R&D investments have a negative impact on firm performance and increase the future volatility of a firm's value.

In spite of the growing support of a relationship between these two constructs, the findings are not uniform across studies. Some researchers have suggested that one of the limitations in these studies is the lack of consideration of a time lag. The innovation process is of a cumulative nature (51), which indicates lag effects of R&D investments. R&D is a long horizon investment, requiring a long period to realize payoffs, especially for pharmaceutical firms (52, 53). Previous studies have shown that, on average, the time lag is 2 years for electrical machinery and metal manufacturing, 5 years for pharmaceutical manufacturing, and 3 years for the remaining industries (54).

R&D investments normally take a certain amount of time to achieve an economic effect on the market value of a firm, especially in the pharmaceutical industry, for several reasons. First, a new drug must go through preclinical and clinical trials, which is a long-term process that takes approximately 8.3 years (55, 56). Second, unlike other consumer goods, a new drug must receive approval from the Food and Drug Administration (FDA) to ensure drug safety, efficacy, quality, and accessibility, which is complex and takes time (57). Third, after the success of research and development, a series of processes are needed to truly transform the success of research and development into actual economic benefits, such as applying for patents to protect a firm's R&D results from being stolen by competitors, which is time consuming (58). Therefore, the immediate financial impact of R&D investment might not be positive if the premium benefits for those innovative products are not enough to offset the costs.

Although a few studies have noted that these effects experience a lag in the pharmaceutical industry, most of them have focused on the context of developed countries. For example, Nord (59) found a positive and significant relationship between R&D investments with a lag of 10 years and market value in the pharmaceutical industry from data of the top 16 grossing pharmaceutical companies in the USA. After conducting a study on Korean pharmaceutical firms, Lee and Choi (13) concluded that there is a significantly positive relationship between R&D intensity of the previous 2 and 5 years and a firm's value. Based on a finite distributed lag model, Karpa and Nowakowski (60) found that there is a 2-year lag between R&D investments and firm performance in the European healthcare industry.

However, there are extremely significant differences between the pharmaceutical industries of China and developed countries. First, the drug approval and new drug registration times in China are often prolonged due to China's regulatory standards, which are inconsistent with international practices, lack sufficient manpower in terms of the Center for Drug Evaluation (CDE), and involve excessive applications of generic drug products (26). Second, the high fragmentation of the industrial structure driven by local protectionism is a hallmark of China's pharmaceutical industry (25, 26). Dispersed innovation resources and weak R&D infrastructure caused by this high fragmentation contribute to low returns on R&D due to the long process of new drug development (25). Third, the Chinese government has implemented tax preferences and subsidies to encourage independent innovations in the pharmaceutical industry (25). Such subsidies and tax preference influence the returns of R&D investment by reducing the costs and uncertainty associated with innovation, integrating innovation resources, dispersing enterprise R&D risks, and reducing the financing costs (61–63). Therefore, whether there is a lagged effect between R&D investments and firm performance in China's pharmaceutical firms is largely unexplored.

In summation, R&D investments are highly resource consuming and may have a negative impact on a firm's performance. However, in the long run, firms' R&D achievements can bring them economic benefits and technological advantages, bring long-term economic benefit growth, and finally bring positive impacts on firm performance (48). Based on the above analysis, we propose the following research hypotheses:

**Hypothesis 1a: R&D investments are negatively related to current firm performance**.
**Hypothesis 1b: One-year lagged R&D investments are positively related to current firm performance**.
**Hypothesis 1c: Two-year lagged R&D investments are positively related to current firm performance**.

### The Moderating Role of Debt Capital

Debt is a critical source of funds for most Chinese firms, accounting for over 90% of all external fund financing (19, 64, 65). A recent debate exists on the benefits and costs of debt on R&D investments (19, 34, 66, 67). R&D investments are generally resource consuming (68). As such, scholars have argued that a firm with a high level of R&D investment may need to reduce other financial obligations so as to mitigate its risk of financial distress (69). We argue that debt provides inappropriate governance for R&D in China's pharmaceutical firms for several theoretical reasons.

First, pharmaceutical R&D investments inherently involve a higher degree of information asymmetry, and therefore cause a serious “lemon” problem (70). In particular, in emerging economies such as China, creditors cannot always effectively monitor debtors, because lending transactions may not be based entirely on an arms-length basis, and the legal protection may not be well defined or fully enforced (29, 71). Therefore, credit institutions may charge higher interest premiums (72), thus increasing R&D expenses.

Second, according to transaction cost economics (TCE), the asset specificity of R&D investments may hinder firms' access to debt financing (20). New drug R&D projects involve intangible research capabilities, and such capabilities are firm-specific assets, which have a lower resale value than do general assets (73, 74). As investments in R&D involve firm-specific assets that serve as poor collateral, lenders of debt are reluctant to fund such investments (20, 75, 76) or require higher interest premiums.

Third, prior research has concluded that debt provides inappropriate governance safeguards for R&D investments, and empirical tests have shown that debt and R&D intensity are negatively associated (76–78). Based on the above discussion, we propose the following hypothesis:

**Hypothesis 2: The negative relationship between R&D investments and firm performance will be strengthened when the debt capital is high**.

### The Moderating Role of Ownership Concentration

Ownership “represents a source of power that can be used to either support or oppose management depending on how it is concentrated and used” (79). Concentrated ownership is common around the world, especially in emerging economies (80–82). The negative effect of R&D investments on firm performance is amplified by the level of concentrated ownership in China's pharmaceutical industry for several theoretical reasons.

First, from the principal–principal (PP) perspective, concentrated ownership, together with weak institutions, has been identified as the “root cause” of PP conflicts, defined as the goal incongruence among shareholder groups in a firm, particularly between the controlling and minority shareholders (27, 28, 83). PP conflicts are more likely in emerging economies, such as China, which are generally characterized by weak protection for minority shareholders (84, 85). Such conflicts can potentially result in controlling shareholders' expropriation, tunneling behaviors, and, thus, engaging in non-value investments for personal benefits (27, 28, 83).

Second, from the perspective of corporate governance, more concentrated ownership, such as that typical of some East Asian countries, might be less prone to R&D investments because it impedes firms from diversifying the risk of a project across a large number of investors (86). Therefore, based on the above discussion, we propose the following hypothesis:

**Hypothesis 3: The negative relationship between R&D investments and firm performance will be strengthened when the ownership concentration is high**.

### The Joint Consideration of Debt Capital, Ownership Concentration, and R&D Investments

R&D investments are highly resource consuming; therefore, reduced financial obligations are very important for firms that invest heavily in R&D (69). As argued in Hypotheses 2 and 3, firms with high levels of R&D investment can achieve higher performance when they have either a dispersed ownership structure or a low debt level. A recent debate exists on whether ownership structure and debt can be considered substitutes from the control of agency problems (87–89) or as complementary, which implies both the monitoring and expropriation of minority shareholders (90–92). However, the organizational decision must address both agency and monitoring problems.

We argue that in China's pharmaceutical industry, R&D-intensive firms with a lower debt level and a more dispersed ownership structure exhibit the strongest growth. Building on previous works, a high concentration of ownership will come together with higher debt to exert a mutual control over management's activities (93–95), especially in countries with a weak financial market infrastructure, as well as a weak enforcement capacity of regulatory and legal institutions, such as China (32, 96). In such circumstances, the rigidity of debt contracts may largely impair the financial flexibility needed to pursue a sustained project of R&D investments (97). Thus, based on the above discussion, we propose the following hypothesis:

**Hypothesis 4: There is a three-way interaction between a firm's R&D investments, debt capital, and ownership concentration, which implies that the relationship between R&D investments and firm performance is strongest when both the debt capital and ownership concentration are low**.

### Data and Methodology

#### Sample Selection and Data Sources

We tested our hypotheses in the context of the pharmaceutical industry in China from 2009 to 2018. We chose this industry primarily because it is an innovation-driven industry with a higher innovation investment ratio (98), which plays an important role in China's national economy. By using a dataset on Chinese A-share companies in the pharmaceutical industry listed on the Shanghai and Shenzhen stock exchanges, we adopted a three-stage process to determine the final sample. First, we excluded those firms listed with ST and ^*^ST, which indicate their abnormal financial condition and withdrawal risk, respectively (99). Second, we excluded those firms that did not have complete R&D investment records for at least 5 years to control for short panel bias (100). Third, we excluded firms mainly engaged in Chinese herbal medicine processing and sales, due to the cycle and process of research and development for Chinese herbal medicine differing from other medicine (101). The resulting sample contained 56 listed firms in the pharmaceutical industry from 2009 to 2018. After a listwise deletion of observations with missing data, the effective sample for analysis contained 450 firm-year observations. The main data of this research were obtained from the China Stock Market & Accounting Research (CSMAR) database.

#### Measures

Definitions of the specific variables are shown in Table 1.

**Table 1:** Variable descriptions.

**Variables**	**Descriptions**	**Reference**
**Dependent variable**		
Return on Assets (ROA)	Net income/total assets	(33, 37, 102)
**Independent variable**		
Research and Development investments (R&D)	R&D expenditures/total assets	(19, 41, 105, 106)
**Moderating variables**		
Debt capital (DC)	Total liabilities/total assets	(107–109)
Ownership concentration (OC)	The proportion of equity shares held by the largest three shareholders	(110–112, 146)
**Control variables**		
Firm size (SIZE)	Total assets	(115, 116)
Firm age (AGE)	The number of years since a firm's founding	(117, 118)
State ownership (STATE)	Dummy variable, state = 1, non-state = 0	(119, 120)
Fixed assets turnover (FAT)	Operating income / Average net fixed assets	(123, 124)
Market competition (MC)	The squared sum market shares of all firms operating in an industry for a given year	(125, 126)
Sales growth (GROWTH)	The growth rate of firm sales revenue from year t-1 to year t	(99, 127)

##### Dependent Variable

Following prior research (33, 37, 102), this study used the ROA to measure firm performance. ROA (defined as the net income divided by total assets) is commonly used to assess financial results and is one of the most important indicators of firm performance, which more comprehensively reflects a firm's profit ability and their input–output situation (103, 104).

##### Independent Variable

Following prior studies (19, 41, 105, 106), we measured R&D investments by the ratio of a firm's annual R&D expenditures to its total assets.

##### Moderating Variables

Based on prior studies (107–109), debt capital was measured by the ratio of total debt to total assets. The ownership concentration was measured by the proportion of equity shares held by the largest three shareholders (110–112). We focused on the largest three shareholders, i.e., the controlling shareholders, as opposed to the largest five or 10 shareholders, because they are in a unique position to expropriate from other shareholders in the Chinese context (113, 114).

##### Control Variables

Consistent with prior research in this area and the effort to exclude alternative explanations, this study included the following control variables: Firm size, fixed asset turnover, market competition, firm age, state ownership, and sales growth. Given that larger firms may have more favorable access to capital and more resources to generate high performance than smaller firms (115, 116), we controlled for the firm size, as measured by the natural logarithm of the total assets. We operationalized firm age, which may account for a firm's experience (117, 118), as the number of years since a firm was founded. State ownership may influence firm performance through outsider support (119, 120); therefore, we controlled for state ownership, as measured by a dummy variable, taking the value of “1” if a firm is a state-owned, and “0” otherwise. Firms with a higher fixed asset turnover have higher production efficiency and better performance (121, 122); therefore, this study controlled for fixed asset turnover, as measured by dividing the operating income by the average net fixed assets (123, 124). Given that the intensity of market competition may affect the business strategy and market resource allocation of firms (125, 126), we controlled for the market competition, measured as the Herfindahl–Hirschman Index (HHI), as constructed by adding the squared market shares of all firms operating in an industry for a given year. Furthermore, we included sales growth, which is measured by the growth rate of a firm's sales revenue from year *t*−1 to year *t* (99, 127).

#### Equations

A multivariate linear regression model was constructed to verify the time lag effect of R&D investments on firm performance, as per the following model (1).


(1) $$\begin{array}{l} ROA_{it}=\alpha_{0}+\alpha_{1}R\&D_{it}+\alpha_{2}R\&D_{i(t-1)}+\alpha_{3}R\&D_{i(t-2)}\\ \;+\alpha_{4}R\text{\&}D_{i(t-3)}+\alpha_{5}SIZE_{it}+\alpha_{6}FAT_{it}+\alpha_{7}STATE_{it}\\ \;+\alpha_{8}MC_{it}+\alpha_{9}AGE_{it}+\alpha_{10}GROWTH_{it}+\varepsilon_{it} \end{array}$$

In model (1), α_0_ is a constant term, the coefficients α_1_ and α_4_ are the coefficients of R&D from the current year to the 3-year lag used to capture the lag effect of R&D investments on firm performance, and ε_*it*_ is an ordinary error term. The control variables include firm size (SIZE), fixed assets turnover (FAT), market competition (MC), firm age (AGE), sales growth (GROWTH), and state ownership (STATE).

On the basis of model (1), to evaluate the moderating role on firm performance, we formulated the following model (2).


(2) $$\begin{array}{l} ROA_{it}\;=\beta_{0}+\beta_{1}R\&D_{it}+\beta_{2}DC_{it}\;+\beta_{3}OC_{it}+\beta_{4}{\left(R\&D^{*}DC\right)}_{it}\\ \;\beta_{5}{\left(R\&D^{*}OC\right)}_{it}++\beta_{6}{\left(DC^{*}OC\right)}_{it}+\beta_{7}{\left(R\&D^{*}DC^{*}OC\right)}_{it}\\ \;+\beta_{8}Control_{it}+\;\varepsilon_{it} \end{array}$$

In model (2), β_0_ is a constant term, the coefficients β_4_ and β_5_ are the coefficients of the interaction terms used to capture the moderating role of debt capital (DC) and ownership concentration (OC), the coefficient β_7_ is the coefficient of the three-way interaction terms of R&D, DC, and OC, and ε_*it*_ is an ordinary error term.

## Results

Tables 2, 3 report the means, standard deviations, correlations, and variance inflation factors (VIFs) of all of the variables used in the analyses. To address potential multicollinearity, we calculated the VIFs for all of the predictors in the model. All VIFs associated with each predictor were within the range of 1.02–1.70, with a mean of 1.27. These results are well within acceptable limits, suggesting that multicollinearity is not a concern (128, 129).

**Table 2:** Descriptive statistics.

**Variable**	**Obs**	**Mean**	**Std. Dev**.	**Min**	**Max**
ROA	450	0.057	0.046	−0.148	0.219
R&D	450	0.045	0.152	0	1.926
DC	450	0.751	0.763	0.016	7.575
OC	450	0.449	0.142	0.129	0.873
SIZE	450	15.535^a^	7.244^a^	5.865^a^	51.457^a^
AGE	450	16.602	4.867	2	27
STATE	450	0.253	0.435	0	1
FAT	450	2.832	2.332	0.298	24.313
MC	450	0.03	0.006	0.025	0.05
GROWTH	450	0.21	0.545	−0.425	9.85

a * In units of thousand*.

**Table 3:** Correlation matrix.

		**VIF**	**1**	**2**	**3**	**4**	**5**	**6**	**7**	**8**	**9**	**10**
1	ROA		1									
2	R&D	1.05	−0.176***	1								
3	OC	1.34	0.308***	−0.134***	1							
4	DC	1.36	−0.329***	−0.028	−0.125***	1						
5	SIZE^a^	1.70	−0.125***	0.026	0.158***	0.343***	1					
6	STATE	1.25	−0.055	−0.095**	0.155***	0.328***	0.274***	1				
7	FAT	1.39	0.490***	−0.090**	0.293***	−0.343***	−0.344***	−0.025	1			
8	MC	1.02	0.041	−0.036	0.060	0.019	−0.104**	0.033	0.042	1		
9	AGE^a^	1.27	−0.178***	−0.046	−0.155***	0.187***	0.393***	0.018	−0.095**	−0.136***	1	
10	GROWTH	1.02	0.152***	−0.036	0.052	0.004	0.052	−0.060	−0.029	0.011	-0.044	1

a * Logarithmic form*.

*** *p < 0.01*,

** *p < 0.05*,

*p < 0.1.

We ran a Hausman test (130) to check whether random- or fixed-effects models were more appropriate for this panel set. The results of the Hausman test indicate that the fixed effects model is more robust for the case of the regression specifications with the current panel data (*p* < 0.1). Tables 4, 5 report the results of the panel regression analysis.

**Table 4:** Fixed-effects analyses of R&D investments on ROA.

	**Model 1^a1^**	**Model^a2^**	**Model 3^a3^**	**Model 4^a4^**	**Model 5^a5^**
SIZE^a^	0.001	−0.002	0.005	0.013	0.005
	(0.017)	(0.018)	(0.018)	(0.019)	(0.022)
STATE	0.004	0.007	0.004	0.002	0.005
	(0.009)	(0.009)	(0.009)	(0.008)	(0.008)
FAT	0.004*	0.004*	0.004**	0.004	0.002
	(0.002)	(0.002)	(0.002)	(0.002)	(0.002)
MC	0.057	0.035	0.114	−0.098	−0.169
	(0.173)	(0.172)	(0.192)	(0.290)	(0.325)
AGE^a^	−0.033	−0.024	−0.035*	−0.069**	−0.055*
	(0.020)	(0.021)	(0.021)	(0.026)	(0.028)
GROWTH	0.010	0.010	0.009	0.008	0.007
	(0.007)	(0.007)	(0.007)	(0.007)	(0.007)
R&D_(t)_		−0.055***			
		(0.008)			
R&D_(t−1)_			−0.016***		
			(0.004)		
R&D_(t−2)_				0.061***	
				(0.007)	
R&D_(t−3)_					0.040***
					(0.011)
Constant	0.123	0.133	0.087	0.118	0.163
	(0.130)	(0.131)	(0.131)	(0.140)	(0.180)
Observations	450	450	422	367	313
R^2^	0.106	0.134	0.108	0.124	0.060
Adjusted R^2^	0.094	0.120	0.093	0.107	0.038

a * Logarithmic form*.

a1 * Hausman-test: χ(7)^2^ =22.32, p = 0.0022*.

a2 * Hausman-test: χ(8)^2^ =22.19, p = 0.0046*.

a3 * Hausman-test: χ(8)^2^ =26.73, p = 0.0008*.

a4 * Hausman-test: χ(8)^2^ = 42.33, p = 0.0000*.

a5 * Hausman-test: χ(8)^2^ = 35.50, p = 0.0000*.

*Robust standard errors in parentheses*.

*** *p < 0.01*,

** *p < 0.05*,

* *p < 0.1*.

**Table 5:** Moderating effect of debt capital and ownership concentration on ROA.

	**Model 1^b1^**	**Model 2^b2^**	**Model 3^b3^**	**Model 4^b4^**	**Model 5^b5^**	**Model 6^b6^**	**Model 7^b7^**
SIZE^a^	−0.004	−0.001	−0.006	−0.002	0.005	0.007	0.002
	(0.016)	(0.015)	(0.015)	(0.016)	(0.017)	(0.017)	(0.018)
STATE	0.007	0.006	0.009	0.007	0.004	0.004	0.007
	(0.008)	(0.008)	(0.008)	(0.008)	(0.008)	(0.008)	(0.008)
FAT	0.003	0.002	0.003	0.002	0.004**	0.004**	0.004*
	(0.002)	(0.002)	(0.002)	(0.002)	(0.002)	(0.002)	(0.002)
MC	0.055	0.024	0.042	0.026	0.123	0.126	0.103
	(0.176)	(0.180)	(0.179)	(0.183)	(0.195)	(0.195)	(0.195)
AGE^a^	−0.021	−0.025	−0.017	−0.023	−0.032	−0.031	−0.022
	(0.021)	(0.020)	(0.021)	(0.022)	(0.022)	(0.022)	(0.023)
GROWTH	0.010	0.010	0.010	0.009	0.010	0.009	0.011
	(0.007)	(0.007)	(0.006)	(0.006)	(0.007)	(0.006)	(0.007)
R&D_(t)_	−0.052***	−0.055***	−0.120***	−0.070*			
	(0.009)	(0.009)	(0.039)	(0.038)			
DC_(t)_	−0.010*	−0.017***	−0.009*	−0.015**			
	(0.005)	(0.005)	(0.005)	(0.007)			
OC_(t)_	0.030	0.038	0.032	0.044			
	(0.040)	(0.039)	(0.040)	(0.040)			
${\text{R}\&\text{D}}_{\left(\text{t}\right)}^{*}$DC_(t)_		−0.231***		−0.060			
		(0.060)		(0.100)			
${\text{R}\&\text{D}}_{\left(\text{t}\right)}^{*}$OC_(t)_			−0.335*	−0.085			
			(0.172)	(0.161)			
${\text{DC}}_{\left(\text{t}\right)}^{*}$OC_(t)_				−0.001			
				(0.034)			
${\text{R}\&\text{D}}_{\left(\text{t}\right)}^{*}$${\text{DC}}_{\left(\text{t}\right)}^{*}$OC_(t)_				1.236**			
				(0.517)			
R&D_(t−1)_					−0.015***	−0.018***	−0.122**
					(0.004)	(0.004)	(0.056)
DC_(t−1)_					−0.002	−0.012***	−0.002
					(0.004)	(0.004)	(0.003)
OC_(t−1)_					0.021	0.034	0.021
					(0.041)	(0.041)	(0.041)
${\text{R}\&\text{D}}_{\left(\text{t}-1\right)}^{*}$DC_(t−1)_						−0.297***	
						(0.073)	
${\text{R}\&\text{D}}_{\left(\text{t}-1\right)}^{*}$OC_(t−1)_							−0.535**
							(0.261)
Constant	0.147	0.121	0.145	0.129	0.079	0.060	0.081
	(0.115)	(0.113)	(0.114)	(0.113)	(0.128)	(0.124)	(0.129)
Observations	450	450	450	450	422	422	422
R^2^	0.160	0.202	0.168	0.222	0.111	0.158	0.132
Adjusted R^2^	0.143	0.184	0.149	0.199	0.0913	0.138	0.111

a * Logarithmic form*.

b1 * Hausman-test: χ(10)^2^ =20.67, p = 0.0235*.

b2 * Hausman-test: χ(11)^2^ = 19.54, p = 0.0520*.

b3 * Hausman-test: χ(11)^2^ = 21.82, p = 0.0528*.

b4 * Hausman-test: χ(14)^2^ = 23.23, p = 0.0567*.

b5 * Hausman-test: χ(10)^2^ = 25.00, p = 0.0053*.

b6 * Hausman-test: χ(11)^2^ = 21.02, p = 0.0331*.

b7 * Hausman-test: χ(11)^2^ = 26.19, p = 0.0061*.

*Robust standard errors in parentheses*.

*** *p < 0.01*,

** *p < 0.05*,

* *p < 0.1*.

Table 4 presents the hierarchical regression results used to examine Hypotheses 1a–c. Model 1 is the basic model, including only the control variables, while models 2–5 add the independent variable ranging from the current year to the 3-year lag, respectively. Model 2 tests Hypothesis 1a, which proposed that R&D investments will negatively affect firm performance. Model 2 shows that the coefficient of R&D is negative and statistically significant (*b* = −0.055; *p* < 0.01), supporting Hypothesis 1a. Hypothesis 1b predicted that R&D investments will have a 1-year lagged positive effect on performance. In model 3, the coefficient for R&D in year *t*−1 is negative and significant (*b* = −0.016; *p* < 0.01); therefore, Hypothesis 1b is not supported. In model 4, the coefficient for R&D in year *t*−2 (*b* = 0.061; *p* < 0.01) is positive and significant, supporting Hypothesis 1c.

**Figure 1:**
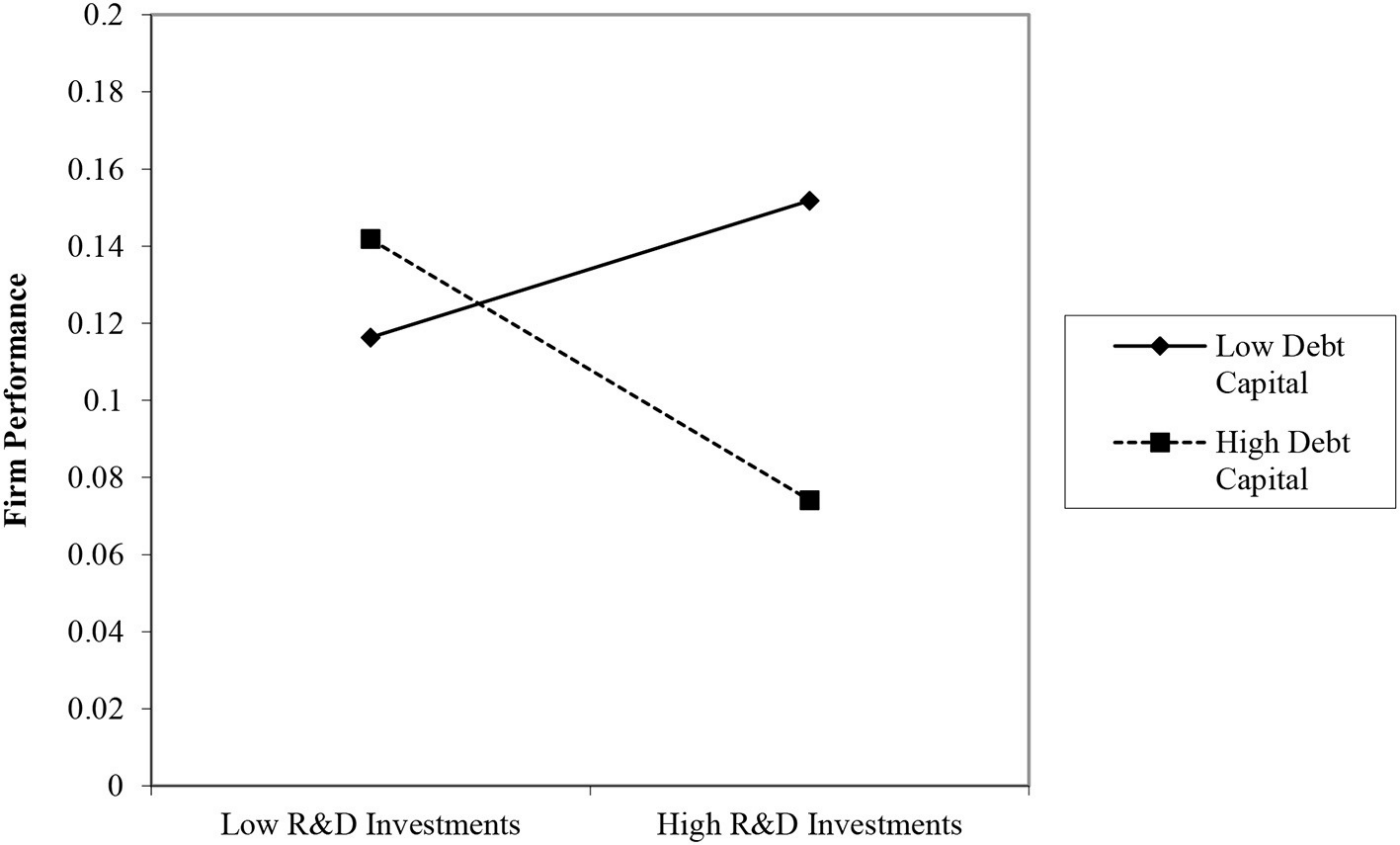
Moderating role of debt capital on the relationship between R&D investments and firm performance.

**Figure 2:**
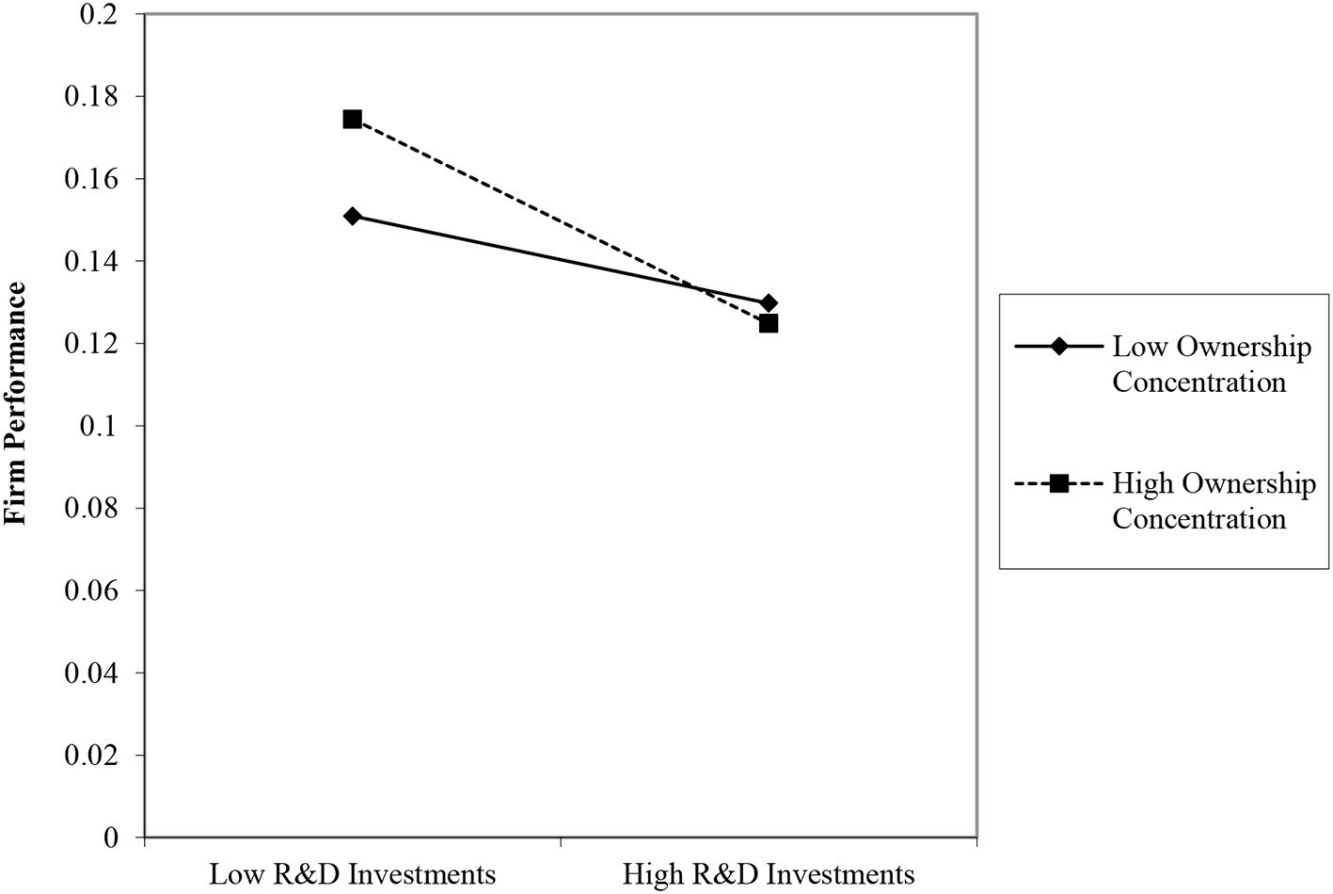
Moderating role of ownership concentration on the relationship between R&D investments and firm performance.

Table 5 presents the hierarchical regression results used to examine Hypotheses 2–4. Model 1 is the basic model that includes the independent, moderating, and control variables. Models 2–4 incorporate interaction effects. Hypothesis 2 proposed that DC will strengthen the impact of R&D investments on performance. The significant and negative coefficient in model 2 (*b* = −0.231; *p* < 0.01) provides strong support for Hypothesis 2. As shown in the plot in Figure 1, the negative relationship between R&D investments and firm performance is stronger when debt capital is higher. Hypothesis 3 predicted a negative moderating effect of OC on the R&D–performance relationship. The interaction term in model 3 reveals that this effect is negative and significant (*b* = −0.335; *p* < 0.1), supporting Hypothesis 3. As shown in the plot in Figure 2, R&D investments have a stronger negative relationship with firm performance when the level of concentrated ownership is high.

**Figure 3:**
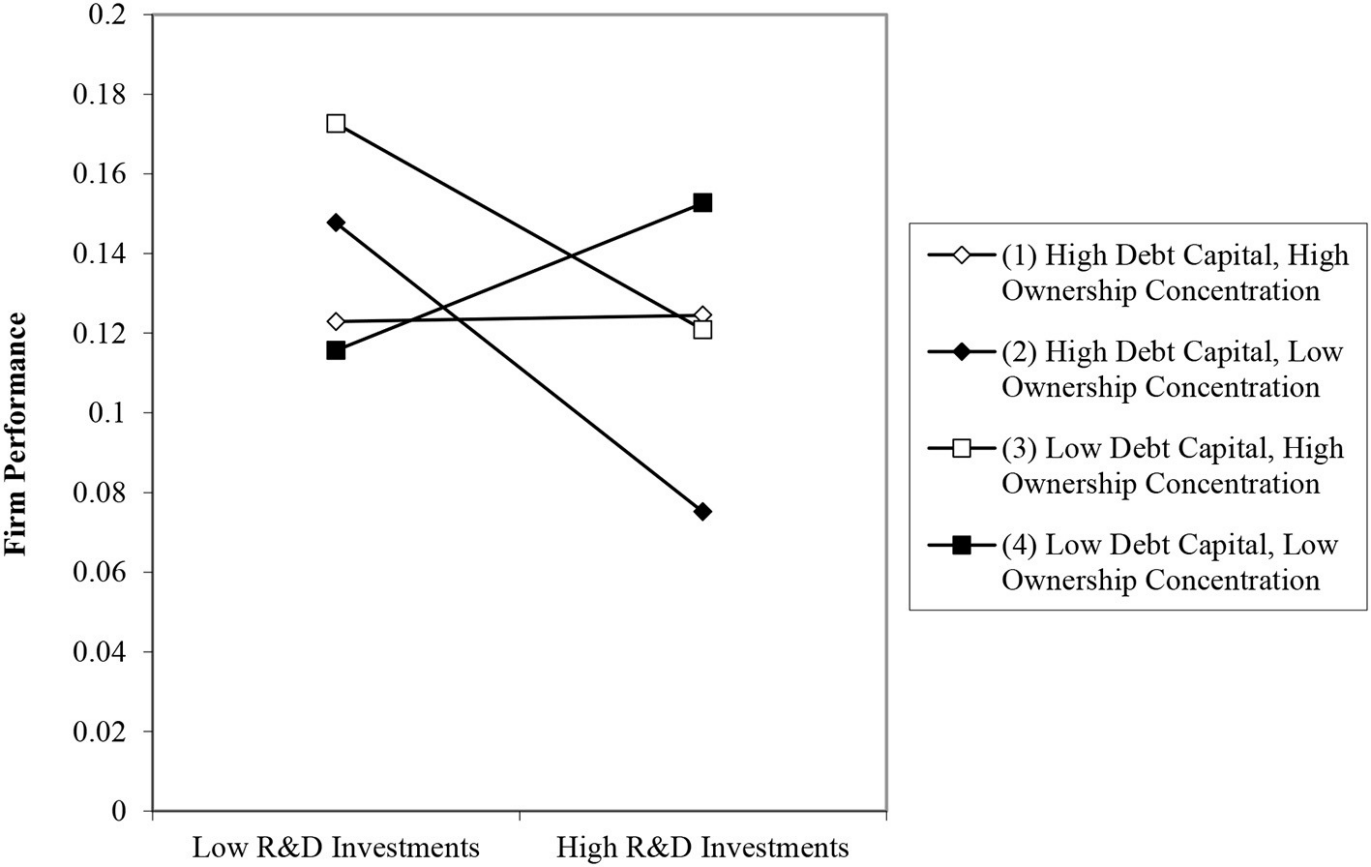
Three-way interactive effects of R&D investments, debt capital and ownership concentration on firm performance.

Finally, model 4 in Table 5 includes the hypothesized three-way interaction term for R&D investments, debt capital, and ownership structure. This three-way interaction is plotted in Figure 3 following the procedure outlined by Cohen (131). The results of slope difference tests for the three-way interaction are shown in Table 6. To identify and interpret a three-way interactive effect, it is necessary to enter all of the two-way interactions, along with the three-way interaction (132, 133). In support of Hypothesis 4, the three-way interaction coefficient is positively significant (model 4 in Table 5; β = 1.236, *p* < 0.05), and the change in *R*^2^ indicates a significant improvement in the model fit over models 2–4 in Table 5. We conducted slope difference tests to determine whether the individual slopes were statistically different from one another (134).

**Table 6:** Slop different test.

**Pair of slopes**	***t*-value for slop different**	***p*-value for slop different**
(1) and (2)	1.725	0.085
(1) and (3)	0.678	0.498
(1) and (4)	−0.889	0.374
(2) and (3)	−0.371	0.711
(2) and (4)	−8.038	0.000
(3) and (4)	−3.008	0.003

### Robustness Test

We ran a number of tests to check the robustness of the results. We first tested our models with a 1-year lagged independent variable and the related moderating effects; the results remained unchanged, as shown in models 6 and 7 in Table 5. Second, we checked whether the results were sensitive to different measures of firm performance. Following prior studies (99, 135, 136), we used the return on equity (ROE) as an alternative variable of firm performance. The results of models 2–4 in Table 7, in which firm performance is measured as ROE, show that the coefficient for current and 1-year lagged R&D investments is negative and significant (models 2 and 3; *p* < 0.01), while the coefficient for 2-year lagged R&D investments is positive and significant (model 4; *p* < 0.01). Accordingly, the findings support Hypotheses 1a–c. For the moderating effect of debt capital, the results of model 2 in Table 8 show that the interaction of R&D investments and debt capital has a negative and significant effect on firm performance (*p* < 0.05). Therefore, Hypothesis 2 is supported. Regarding the moderating effect of the ownership concentration, the results of model 3 in Table 8 show that the interaction of R&D investments and the ownership concentration has a negative and significant effect on firm performance (*p* < 0.1), supporting Hypothesis 3. For the three-way interaction between a firm's R&D investments, debt capital, and ownership concentration, the results of model 4 in Table 8 show that the interaction of the three variables is positive and significant (*p* < 0.01), supporting Hypothesis 4. The results in Tables 7, 8, in which new firm performance is measured as ROE, are also similar to those reported in Tables 4, 5. In summation, these results are consistent with our main regressions.

**Table 7:** Fixed-effects analyses of R&D investments on ROE.

	**Model 1^c1^**	**Model 2^c2^**	**Model 3^c3^**	**Model 4^c4^**	**Model 5^c5^**
SIZE^a^	0.012	0.007	0.015	0.025	0.015
	(0.027)	(0.027)	(0.028)	(0.030)	(0.036)
STATE	0.006	0.010	0.009	0.001	0.004
	(0.015)	(0.015)	(0.015)	(0.015)	(0.014)
FAT	0.001	0.001	0.001	−0.001	−0.002
	(0.002)	(0.002)	(0.002)	(0.002)	(0.002)
MC	0.066	0.032	0.202	−0.206	−0.243
	(0.254)	(0.255)	(0.276)	(0.429)	(0.533)
AGE^a^	−0.050*	−0.036	−0.045	−0.115***	−0.101**
	(0.030)	(0.030)	(0.030)	(0.042)	(0.047)
GROWTH	0.018	0.017	0.017	0.014	0.010
	(0.012)	(0.012)	(0.012)	(0.012)	(0.011)
R&D_(t)_		−0.080***			
		(0.012)			
R&D_(t−1)_			−0.039***		
			(0.006)		
R&D_(t−2)_				0.089***	
				(0.013)	
R&D_(t−3)_					0.069***
					(0.017)
Constant	0.103	0.118	0.053	0.186	0.241
	(0.204)	(0.205)	(0.211)	(0.215)	(0.279)
Observations	450	450	422	367	313
R^2^	0.047	0.066	0.050	0.089	0.049
Adjusted R^2^	0.0341	0.0511	0.0341	0.0714	0.0275

a * Logarithmic form*.

c1 * Hausman-test: χ(7)2 = 19.19, p = 0.0076*.

c2 * Hausman-test: χ(8)2 = 19.94, p = 0.0106*.

c3 * Hausman-test: χ(8)2 = 25.45, p = 0.0013*.

c4 * Hausman-test: χ(8)2 = 45.96, p = 0.0000*.

c5 * Hausman-test: χ(8)2 = 37.49, p = 0.0000*.

*Robust standard errors in parentheses*.

*** *p < 0.01*,

** *p < 0.05*,

* *p < 0.1*.

**Table 8:** Moderating effect of debt capital and ownership concentration on ROE.

	**Model 1^d1^**	**Model 2 ^d2^**	**Model 3 ^d3^**	**Model 4 ^d4^**
SIZE^a^	0.002	0.008	0.001	0.004
	(0.024)	(0.024)	(0.023)	(0.024)
STATE	0.011	0.010	0.013	0.009
	(0.016)	(0.017)	(0.016)	(0.017)
FAT	0.000	−0.000	0.000	0.002
	(0.002)	(0.001)	(0.002)	(0.002)
MC	0.044	−0.001	0.028	0.109
	(0.268)	(0.277)	(0.273)	(0.274)
AGE^a^	−0.029	−0.034	−0.023	−0.043
	(0.031)	(0.032)	(0.031)	(0.032)
GROWTH	0.018	0.017	0.018	0.016
	(0.011)	(0.012)	(0.011)	(0.010)
R&D_(t)_	−0.078***	−0.081***	−0.170***	−0.087
	(0.013)	(0.013)	(0.061)	(0.058)
DC_(t)_	−0.005	−0.016	−0.005	0.008
	(0.008)	(0.010)	(0.008)	(0.012)
OC_(t)_	0.046	0.058	0.049	0.058
	(0.067)	(0.066)	(0.066)	(0.069)
${\text{R}\&\text{D}}_{\left(\text{t}\right)}^{*}$DC_(t)_		−0.338**		−0.064
		(0.142)		(0.141)
${\text{R}\&\text{D}}_{\left(\text{t}\right)}^{*}$OC_(t)_			−0.455*	−0.038
			(0.264)	(0.240)
${\text{DC}}_{\left(\text{t}\right)}^{*}$OC_(t)_				0.170**
				(0.068)
${\text{R}\&\text{D}}_{\left(\text{t}\right)}^{*}$${\text{DC}}_{\left(\text{t}\right)}^{*}$OC_(t)_				2.280***
				(0.749)
R&D_(t−1)_				
DC_(t−1)_				
OC_(t−1)_				
${\text{R}\&\text{D}}_{\left(\text{t}-1\right)}^{*}$DC_(t−1)_				
${\text{R}\&\text{D}}_{\left(\text{t}-1\right)}^{*}$OC_(t−1)_				
Constant	0.137	0.100	0.135	0.157
	(0.185)	(0.181)	(0.183)	(0.184)
Observations	450	450	450	450
R^2^	0.071	0.099	0.075	0.134
Adjusted R^2^	0.0517	0.0787	0.0544	0.108

a * Logarithmic form*.

d1 * Hausman-test: χ(10)^2^ =29.34, p = 0.0011*.

d2 * Hausman-test: χ(11)^2^ = 17.59, p = 0.0915*.

d3 * Hausman-test: χ(11)^2^ = 17.88, p = 0.0845*.

d4 * Hausman-test: χ(14)^2^ = 25.50, p = 0.0300*.

*Robust standard errors in parentheses*.

*** *p < 0.01*,

** *p < 0.05*,

* *p < 0.1*.

## Discussion and Conclusion

R&D investments play an important role in sustainable competitive advantages for pharmaceutical firms, especially after COVID-19; however, there is no general consensus in the literature about the effect of R&D investments on firm performance (8, 37, 137). Such mixed findings can contribute to the lack of consideration of time lag effects, and the existence of contingencies that moderate the main effect (8). This study presented a conceptual model by examining the lag effects of R&D activity on firm performance, and the role of financial governance (debt capital and ownership concentration) in the R&D investments–firm performance relationship. In particular, we employed a configurational model to investigate the interaction of three constructs, namely, R&D investments, debt capital, and ownership concentration, that jointly predict different financial outcomes.

The present study makes several important contributions to the existing literature. First, this study contributes to R&D management research by examining the lag effects of R&D investments. Even though the importance of R&D has been reported in multiple studies, the empirical findings of the effects of R&D investments on firm performance are inconsistent (8, 37, 137). We found that the effect of R&D on growth begins in the second year after R&D spending and increases thereafter in China's pharmaceutical industry. The results indicate that returns of R&D investments have a long-term characteristic (138, 139).

Second, this study advances the financial governance literature by highlighting the role of debt capital and ownership concentration on the relationship between R&D investments and firm performance. There are two key issues in firms' R&D investment decisions, namely, the financing of R&D and firms' governance mechanism (19, 20). However, prior studies have focused on factors such as firm size, advertising activity, industry context, and country context (9–14), with less attention being paid to how financial governance in terms of debt and ownership structure affects the returns of R&D. The results showed that a firm's debt and ownership structure can help to mitigate the negative impacts of R&D investments on firm performance.

Third, compared to prior studies exclusively focused on either debt or the equity financing mechanism (77, 78, 97, 140), this study bridges the gap by identifying which level of debt capital and ownership concentration are appropriate for the efficacy of R&D investments. Debt and equity are regarded as two critical sources of funding; however, there is a vigorous debate about the choices between debt and equity financing (141). We found a significant three-way interaction, such that firms that invest heavily in R&D achieve their highest performance when their use of debt capital and their extent of concentrated ownership are both low, as shown in Figure 3.

Finally, this study extends research on how R&D investments and financial governance in terms of debt capital and ownership concentration influence firm performance in the context of an emerging market economy. Scholars have argued that 49% of empirical studies on the returns of R&D have been conducted in the context of advanced market economies, such as the USA, characterized as well-functioning financial systems and having a diluted ownership (142). Compared to developed countries, transitioning economies such as China have an imperfect capital market, weak IPR and creditor protection, and high financial constraints in general, which may impede firms' R&D activities and financing (143–145). Thus, this study contributes to the literature by empirically demonstrating the role of financial governance on the returns of R&D in developing countries.

In summation, the governance implications of debt and the ownership concentration for R&D investments have enormous practical significance for managerial decisions about how best to invest in R&D. In this study, we only lagged the R&D intensity up to 3 years (due to sample limitations). This 3-year window may be too short, given that R&D projects in the pharmaceutical industry may experience longer effects (54). Thus, we encourage researchers with more comprehensive data to explore the patterns of the effects of R&D on different financial choices or in different industries over time.

## Data Availability Statement

The raw data supporting the conclusions of this article will be made available by the authors, without undue reservation.

## Author Contributions

All authors listed have made a substantial, direct and intellectual contribution to the work, and approved it for publication.

## Funding

We gratefully acknowledge the financial support from National Natural Science Foundation of China (grant nos. 71802138 and 71804114) and Ministry of Education in China (grant no. 18YJC630122).

## Conflict of Interest

The authors declare that the research was conducted in the absence of any commercial or financial relationships that could be construed as a potential conflict of interest.

## Publisher's Note

All claims expressed in this article are solely those of the authors and do not necessarily represent those of their affiliated organizations, or those of the publisher, the editors and the reviewers. Any product that may be evaluated in this article, or claim that may be made by its manufacturer, is not guaranteed or endorsed by the publisher.
